# Alcohol Screening and Brief Intervention in Primary Health Care in Kazakhstan—Results of a Cluster Randomised Pilot Study

**DOI:** 10.3389/ijph.2022.1604803

**Published:** 2022-10-10

**Authors:** Uwe Verthein, Harald Lahusen, Marcus Sebastian Martens, Mariya Prilutskaya, Oleg Yussopov, Zhanar Kaliyeva, Bernd Schulte

**Affiliations:** ^1^ Department of Psychiatry and Psychotherapy, University Medical Center Hamburg-Eppendorf, Center for Interdisciplinary Addiction Research, University of Hamburg, Hamburg, Germany; ^2^ Pavlodar Branch of Semey State Medical University, Pavlodar, Kazakhstan; ^3^ Monitoring Center on Alcohol and Drugs, Pavlodar, Kazakhstan; ^4^ Sanjar Dzhafarovich Asfendiyarov Kazakh National Medical University, Almaty, Kazakhstan

**Keywords:** screening, alcohol, primary Health Care, brief intervention, hazardous drinking, risky drinking, AUDIT-C, RE-AIM framework

## Abstract

**Objective:** The aim of this pilot trial was to assess the feasibility of ASBI in primary health care units (PHCUs) in Kazakhstan.

**Methods:** A two-arm cluster randomised trial in five PHCUs based on the RE-AIM framework for implementation studies was carried out. Patients with AUDIT-C scores ≥4 for females and ≥5 for males received a brief face-to-face intervention delivered by a trained physician plus information leaflet (intervention group, IG) or simple feedback including a leaflet (control group, CG).

**Results:** Among 7327 patients eligible for alcohol screening according to the inclusion criteria 1148 patients were screened (15.7%, IG: 11.5%, CG: 27.3%). 12.3% (N = 141) were tested AUDIT-C positive (IG: 9.9%, CG: 15.1%). Out of 112 physicians invited, 48 took part in the ASBI training, 31 finally participated in the study, 21 in the IG (2 PHCUs), 10 in the CG (3 PHCUs). The majority of physicians did not have difficulties in performing the intervention.

**Conclusion:** ASBI is feasible and can be implemented into PHC settings in Kazakhstan. However, the implementation depends on the willingness and interest of the PHCU and the physicians.

## Introduction

Alcohol consumption is a significant risk to public health and a leading cause of morbidity and mortality [1–3]. In addition to diseases in which alcohol consumption is a necessary cause (e.g., alcohol abuse disorders, alcoholic liver disease), alcohol use is causally related to more than 200 ICD-10 diseases, including various cancers, diabetes, cardiovascular disease [1], as well as accidental and intentional injuries [4]. Given this, implementing effective measures to address health and wider societal consequences of alcohol-related harm is a top international public health priority.

There are a number of so-called “best buys” which are recommended by the WHO to reduce harmful use of alcohol. Besides policy interventions such as increasing alcohol taxation, the WHO recommend the widespread implementation of brief psychosocial intervention for persons with hazardous and harmful alcohol use [5]. A prime example for such an intervention is alcohol screening and brief intervention (ASBI) which is especially recommended to be offered by general practitioners (GPs) [6]. Most hazardous and harmful drinkers consult their primary health care (PHC) providers and the greatest impact in addressing alcohol-related harm at a population level can be achieved by focussing on this larger group of hazardous and harmful drinkers. There is large scientific evidence that ASBI is effective and cost-effective in PHC settings [7–10] and some evidence for small effects of ASBI on alcohol consumption reductions in emergency care settings [11].

Screening patients for their alcohol use can be performed *via* conversation or using a formal screening instrument like the AUDIT-C [12, 13], a modification of the Alcohol Use Disorders Identification Test [14]. Brief interventions on alcohol consumption are usually based on the patient’s current motivation and aim to influence the readiness to change in a non-confrontational way [15]. Often, elements of Motivational Interviewing (MI) [16] are used as part of the brief intervention technique [17]. Manuals on brief intervention to support primary care workers have been published by the World Health Organization [18]. Brief interventions can be supplemented by information material such as leaflets or brochures on (risky) alcohol consumption and its consequences.

Several studies have demonstrated the importance of providing training to PHC physicians in order to increase their activity in measuring alcohol use and giving brief advice to heavy drinkers to help reduce their consumption [19–21].

In Kazakhstan, the implementation of a multi-component alcohol prevention policy has been claimed to be a strategic approach in the public health system for over 20 years. It includes age boundaries for sale, taxation and advertisement regulations as well as time and space restrictions on consumption and sale [22]. The measure of pure alcohol per capita is regarded as a generalized indicator of policy effectiveness and its reduction to 6.5 L by 2025 is an aim of the current national strategic programme [23]. Although the PHC settings are claimed to play a pivotal role within the national preventive initiatives, their current alcohol prevention measures represent only a minor part of the comprehensive national screening programme addressing seven lifestyle risk factors for the population aged 30 years and above. For 2016, WHO reported a per capita (age 15+) consumption of 7.7 L of pure alcohol. About a half of those persons aged 15 years or older who had reported to use alcohol in 2016, exhibited heavy episodic drinking behaviour [24].

According to the official register of the Ministry of Health, the incidence of mental and behavioral disorders due to alcohol consumption in the Republic of Kazakhstan was 69.0 per 100,000 inhabitants in 2019 (83.8 in 2018), and 534.0 per 100,000 inhabitants had been under dispensary and consultative observation in the same year (557.1 in 2018) [25]. In most of the cases, people who have hazardous and harmful drinking patterns are registered for dispensary and consultative observation. Compared to the other Central Asian countries, Kazakhstan has exhibited the highest prevalences for both indicators for many years.

Brief interventions have been implemented in Kazakhstan in a number of pilot projects with limited sustainability. The main barriers for ASBI implementation in Kazakhstan were reported to be lack of training, lack of time during patients’ visits, and attitudes towards alcohol consumption in medical personnel [26]. These observations are in accordance with the main barriers of implementation of ASBI found in the international literature: environmental context and resources, beliefs about capabilities, and (lack of) skills [27, 28].

Scientific studies on ASBI have not yet been carried out in Kazakhstan. The primary aim of this pilot cluster-randomised trial was to assess the feasibility of ASBI implementation in primary health care units (PHCU) in Kazakhstan and to compare its efficacy against simple feedback as a control intervention [29].

## Methods

The pilot study was designed as a two-arm cluster randomised trial in order to explore feasibility and acceptability in six PHCUs in Kazakhstan. Every PHCU has its own catchment area. 5 PHCUs were located in Pavlodar City, one PHCU was located in Aksu, about 40 km from Pavlodar (this PHCU dropped out). Therefore, cross-contamination is negligible. In the 5 participating PHCUs in Pavlodar between 38 and 48 physicians treat about 270,000–360,000 patients per year. In the PCHU in Aksu about 90,000 patients are treated by 48 physicians per year. Stratified randomisation was used to allocate three PHCUs to the intervention group (IG) and three PHCUs to the control group (CG). Stratification was based on the assessment of the mean number of patient visits per PHCU per day. Three pairs of PHCUs with comparable doctor-patient ratio were formed. From these pairs, one PHCU was assigned to the IG and one to the CG by the study statistician by means of a random algorithm (using SPSS program syntax [30]). All patients with an appointment in the participating PHCU were eligible for recruitment. Participants had to be aged between 18–69 years, able to follow the study procedures, and have provided written informed consent. Patients with diagnosed lifetime alcohol dependency according to ICD-10 criteria were excluded. The study started in summer 2018, the screening was carried out in 2019. Patients of the IG with an AUDIT-C score of three or lower for females and four or lower for males received short verbal feedback based on their alcohol consumption and an information leaflet reinforcing the benefits of low-risk alcohol use. Patients with higher AUDIT-C scores received a brief (5 min) face-to-face alcohol intervention delivered by a trained PHC physician plus a patient information leaflet. Patients of the CG received simple feedback, including information about their individual AUDIT-C score and the associated alcohol risk level, as well as a patient leaflet with recommendations for low-risk alcohol use. Physicians in the intervention arm received a 3-hour ASBI training plus booster session based on the WHO Europe ASBI training package [31]. The booster group sessions were conducted within six to 8 weeks after the initial training. In the control arm physicians were introduced to the screening procedures with AUDIT-C and AUDIT by the local research staff. The trainers were experienced in Motivational Interviewing (MI) techniques and in providing training courses for health professionals [29].

Patient variables were assessed at two time points, at baseline, concurrently with the screening and brief intervention process, and at 3-month follow-up. The assessments included patient characteristics such as gender, age, and ethnicity, knowledge on alcohol effects and risks, patients’ acceptance and AUDIT-C/AUDIT. Changes in AUDIT-C scores as well as the number of patients who scored AUDIT-C positive at follow-up (≥4 points for females, ≥5 points for males) were analysed between IG and CG by using linear mixed-effects models analysis on an individual level and chi-square-test. The cluster structure was taken into account by nesting patients’ scores within physicians and PHCU. Furthermore, an AUDIT-C change-score for each patient (i.e. the difference between baseline and follow-up score) was calculated, and multivariate testing was carried out using binomial logistic regression in order to analyse the association between patient characteristics and positive AUDIT-C status.

On the provider level, the physicians of the participating PHCU were interviewed pre-training (socio-demographics, experience, attitudes measured by Shortened Alcohol and Alcohol Problems Perception Questionnaire (SAAPPQ)) and post-training (quality of training, SAAPPQ) as well as subsequent to the patient-follow-up period. Providers’ experience was assessed by a list of items such as “How difficult or easy did you find explaining the assessment of alcohol use in terms of standard drinks” or “I found it difficult to deliver ASBI because there was too little time during the patient visit” that should be rated on a 5-point rating scale. This final provider assessment was based on the results of two focus groups with both, participating physicians and physicians who had chosen to decline their participation in the trial. All participating physicians signed an informed consent. Patient variables were assessed within the PHCU, provider assessment took place at the training location and in the PHCU. The study was planned according to the SPIRIT guidelines for protocols of a clinical trial [32]. The statistical analyses were performed by using SPSS 25 [30].

The RE-AIM framework for implementation studies was used to support the evaluation of feasibility and implementation outcomes [33]. Although this trial was directed towards feasibility (based on the five RE-AIM dimensions) the change in AUDIT-C score was chosen as a primary outcome criterion [29]. Outcome measures selected to assess the dimensions Reach and Effectiveness were the rate of eligible patients and the proportions of patients screened and intervened at baseline, and the change in AUDIT-C score between baseline and follow-up. The dimension Adoption included the proportion of staff that participated and their representativeness compared to non-participants. Difficulties and barriers for performing ASBI as well as the acceptance by the patients were the operationalized components of the dimension Implementation. The potential extent to which the intervention package becomes institutionalized and possible facilitators were the factors examined under the Maintenance dimension.

The study was approved by the ethical board of the Kazakh National Medical University in Almaty (application no. 641, IEC session no. 8(72)). Study participation was based on informed consent. The study is registered in the German register of clinical trials (DRKS) at www.drks.de, No. DRKS00015882.

## Results

Deviating from the initial concept, five PHCUs took part, two in the interventional arm and three in the control arm. One PHCU dropped out of the study after the randomisation had taken place. The order of the local health authority was insufficient to motivate this facility to participate in the study. Out of 112 physicians who had originally been invited to participate (56 for both, IG and CG), 24 of each group (42.9%) took part in the training. Screening was performed by 31 physicians (64.6% of physicians trained), 21 of the IG (87.5%) and 10 of the CG (41.7%).

### Reach

**FIGURE 1:**
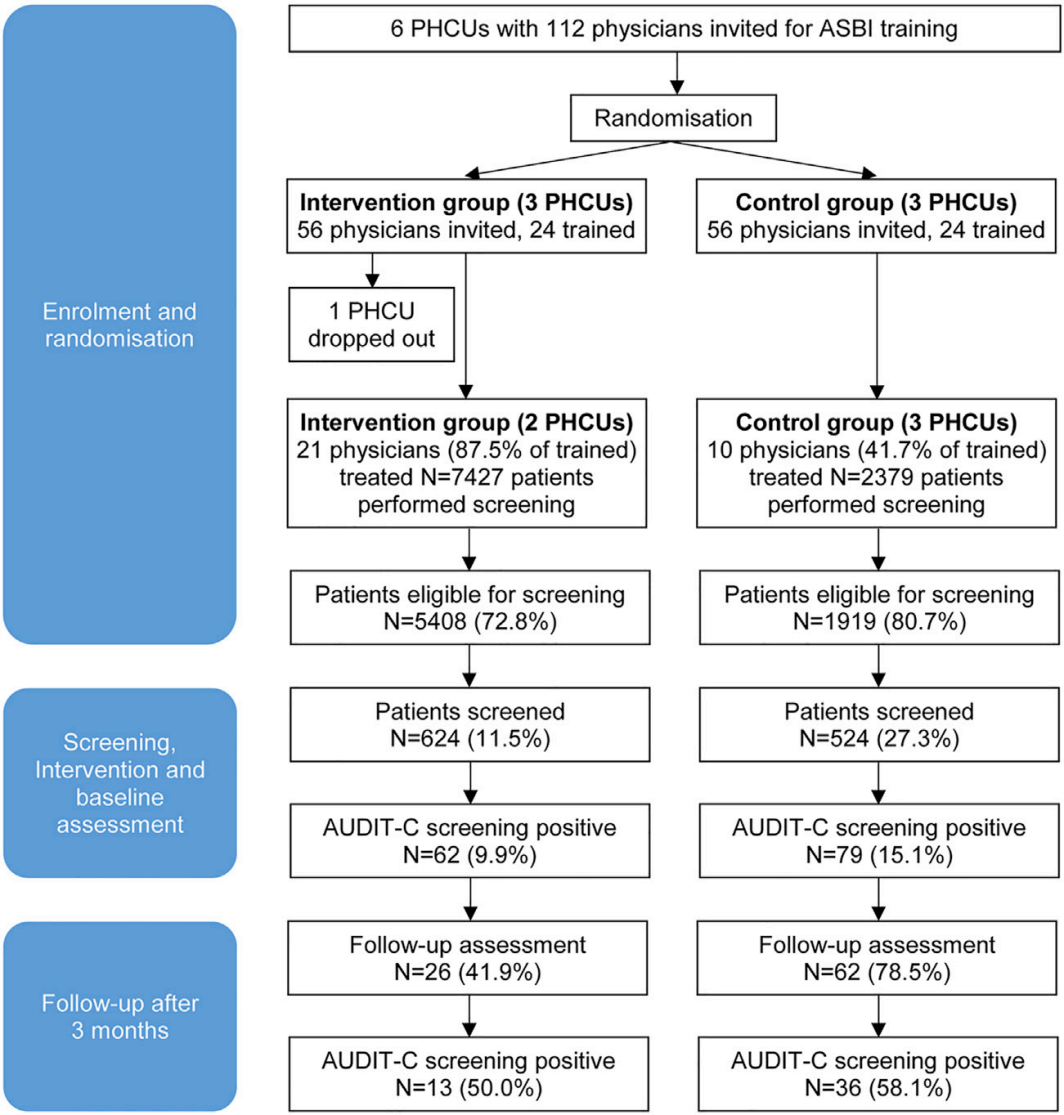
Patient flow in the cluster-randomised trial finally involving 5 primary health care units and 31 physicians (Alcohol Screening and Brief Intervention in Primary Health Care in Kazakhstan—a Cluster randomised Pilot Study, Germany/Kazakhstan, 2022).

Screening was carried out on 139 working days between March and October 2019. In five different PHCUs 9,806 patients were treated by the participating physicians during that period. Among them N = 7,327 patients were eligible for alcohol screening according to the inclusion criteria (74.7%). A number of N = 1,148 patients were screened (15.7%), N = 624 in the IG and N = 524 in the CG (Figure 1).

Overall, 12.3% of the patients screened were tested positive for a hazardous or risky drinking pattern according to the AUDIT-C screening result, corresponding to N = 141 persons; N = 62 (9.9%) in the IG and N = 79 (15.1%) in the CG. Of these patients, N = 88 were reached 3 months later for a follow-up assessment (62.4%). Thirteen patients in the IG (50.0%) and 36 in the CG (58.1%) were AUDIT-C positive at follow-up (Figure 1). The individual reasons for patient drop-out were not assessed due to loss of contact.

At baseline around half of the patients screened in both groups were male (Table 1). The patients in the IG were on average 4 years older than the control patients. About half of the patients in both groups were of Kazakh nationality. The patients in the IG had a higher level of education. Experiences with alcohol related questions and advice showed substantial differences between the study groups; they are less pronounced in the control arm.

**TABLE 1:** Patient characteristics of intervention (Alcohol Use Disorders Identification Test—short form and and brief intervention) and control group (Alcohol Use Disorders Identification Test—short form only) at baseline (Alcohol Screening and Brief Intervention in Primary Health Care in Kazakhstan—a Cluster randomised Pilot Study, Germany/Kazakhstan, 2022).

	Intervention (*n* = 624)	Control (*n* = 524)	Total (N = 1,148)	Significance
Gender				
Male	50.3%	52.1%	51.2%	χ^2^ = 0.37, *p* = 0.545
Female	49.7%	47.9%	48.8%	
Age, years, M (SD)	43.6 (12.8)	39.5 (13.2)	41.7 (13.1)	t = 5.12, *p* = 0.000
Nationality				
Kazakh	52.1%	50.9%	51.5%	χ^2^ = 0.57, *p* = 0.754
Russian	39.3%	39.3%	39.3%	
Other	8.6%	9.8%	9.2%	
Education				
Primary school	2.8%	0.2%	1.6%	χ^2^ = 29.05, *p* = 0.000
High school	53.4%	67.5%	59.9%	
University/academic degree	41.4%	30.5%	36.4%	
Other^a^	2.4%	1.8%	2.1%	
Experience with alcohol related questions and advice past 12 months				
Asked about amount of alcohol	55.5%	10.2%	34.8%	χ^2^ = 254.24, *p* = 0.000
Advised to reduce/stop drinking	46.6%	6.2%	28.1%	χ^2^ = 227.81, *p* = 0.000
Drinking pattern				
AUDIT-C score^b^, M (SD)	1.8 (1.9)	2.2 (2.2)	2.0 (2.1)	t = -3.44, *p* = 0.001
Full AUDIT score, M (SD)	2.9 (3.8)	2.6 (3.4)	2.8 (3.6)	t = 1.24, *p* = 0.216
AUDIT-C = 0	30.4%	25.2%	28.0%	χ^2^ = 8.94, *p* = 0.011
AUDIT-C not positive	59.6%	59.7%	59.7%	
AUDIT-C positive	9.9%	15.1%	12.3%	

^a^ Did not complete high school.

^b^ AUDIT-C: Alcohol Use Disorders Identification Test (short form).

### Effectiveness

The drinking pattern as measured with the AUDIT-C showed small differences between the groups. There were relatively more individuals with an AUDIT-C score of zero in the IG, which corresponds with the lower proportion of AUDIT-C positives. The mean AUDIT-C score in the CG was significantly higher than in the IG (2.2 vs. 1.8; Table 1). The mean scores were far below the threshold score for a risky drinking pattern (which is ≥4 for women and ≥5 for men). The same applies to the full AUDIT score. However, there were no significant group differences here.

Among the 88 positive screened patients reached 3 months later for follow-up assessment, a marked and statistically significant reduction of AUDIT-C scores was observed (Table 2). However, between the IG and CG there was no significant difference as measured by linear mixed-effects models analysis as well as by a comparison of the AUDIT-C change-score between the two groups. With respect to the threshold indicator of the AUDIT-C, exactly half of the IG and 58.1% of the CG were screened positive for a hazardous or risky drinking pattern at time of follow-up. This difference also did not reach statistical significance.

**TABLE 2:** Change in drinking pattern according to Alcohol Use Disorders Identification Test—short form between baseline and 3-month follow-up in intervention (Alcohol Use Disorders Identification Test—short form and brief intervention) and control group (Alcohol Use Disorders Identification Test—short form only) (Alcohol Screening and Brief Intervention in Primary Health Care in Kazakhstan—a Cluster randomised Pilot Study, Germany/Kazakhstan, 2022).

		Intervention	Control	Total	Significance^a^
AUDIT-C score^b^, Median	Baseline	5.0	6.0	6.0	M-W: U = −3.4, *p* = 0.001
	Follow-up	4.0	5.0	4.0	M-W: U = −2.0, *p* = 0.040
AUDIT-C score, IQR	Baseline	3.0	2.3	2.0	
	Follow-up	2.5	2.3	3.0	
AUDIT-C score, M (SD)	Baseline	5.7 (1.7)	6.6 (1.8)	6.3 (1.8)	Time: F = 10.07, *p* = 0.000
	Follow-up	4.1 (2.2)	5.1 (2.1)	4.8 (2.2)	Groups: F = 1.41, *p* = 0.237
AUDIT-C change score		−1.5 (2.4)	−1.4 (1.4)	−1.5 (1.7)	t = −0.20, *p* = 0.839
AUDIT-C positive at Follow-up		50.0%	58.1%	55.7%	OR = 0.72, *p* = 0.487 (95%-CI: 0.29–1.81)
N		26	62	88	

^a^ Time effect and difference between groups. Linear mixed-effects models analysis (time effect nested within physicians and PHCU), IQR: interquartile range, M-W: Mann-Whitney U-test, 95%-CI: 95% confidence interval.

^b^ AUDIT-C: Alcohol Use Disorders Identification Test (short form).

Only a minority of patients followed-up had visited a psychiatrist or narcologist within the past 3 months (total 4.8%), 9.5% of the IG and 3.2% of the CG (χ^2^ = 4.45, *p* = 0.108).

There is no significant association between being AUDIT-C positive at follow-up and the characteristics gender, age, nationality, and academic education in the IG and CG. This applies to bivariate testing as well as to multivariate testing in a logistic regression model (intervention: adjusted OR = 0.66, *p* = 0.396, 95%-CI: 0.25–1.74). Furthermore, better knowledge on alcohol effects and risks is not significantly associated to AUDIT-C result at follow-up, neither in the IG nor in the CG.

### Adoption

Out of 112 physicians originally invited (56 per group, Figure 1), data were available for 99 persons, 31 participated in the trial (27.7%). The participating physicians did not differ significantly from non-participating physicians with regard to gender (participants: 17.4% male, N = 23; non participants: 5.3% male, N = 76; Fisher’s exact test: *p* = 0.082), age (participants: M = 38.1, SD = 13.2, N = 23; non participants: M = 37.4, SD = 13.1, N = 75; Mann-Whitney-Test: U = 823.5, *p* = 0.743), years of work experience (participants: M = 12.9, SD = 12.5, N = 21; non participants: M = 11.9, SD = 12.5, N = 73; Mann-Whitney-Test: U = 722.5, *p* = 0.689) and overall job satisfaction according to the WCW scale [34] (participants: M = 5.06, SD = 1.47, N = 18; non participants: M = 5.13, SD = 1.62, N = 47; Mann-Whitney-Test: U = 407.0, *p* = 0.811). With respect to the SAAPPQ results the attitudes of the physicians towards people with alcohol use disorders did not change significantly between pre and post training (Pre: M = 3.6, SD = 0.9, Post: M = 3.3, SD = 0.8, Wilcoxon-test: Z = -1.67, *p* = 0.095). Furthermore, before and post training there were no significant differences between participants and non participants in the SAAPPQ results (pre: Mann-Whitney-Test: U = 756.0, *p* = 0.473; post: Mann-Whitney-Test: U = 146.0, *p* = 0.124).

### Implementation

The majority of treating physicians in the IG and the CG did not have difficulties in performing ASBI. If at all, bringing up the topic of alcohol in the presence of the patient and referring a patient in case of severe alcohol problems were the most problematic topics in conducting ASBI (Table 3). Feedback and explaining on the risks associated with alcohol use were rated as “quite easy” on average. Thus, these components of ASBI could be implemented in line with the study protocol.

**TABLE 3:** Rating of implementation components of alcohol screening and brief intervention/simple feedback in routine practice. Comparison of intervention and control group (multiple responses, physicians’ perspective) (Alcohol Screening and Brief Intervention in Primary Health Care in Kazakhstan—a Cluster randomised Pilot Study, Germany/Kazakhstan, 2022).

	Intervention		Control		Total	
ASBI components, difficulty	Mean^a^	difficult (4/5)	Mean	difficult (4/5)	Mean	difficult (4/5)
Bringing up the topic of alcohol	2.5	0.0%	3.0	20.0%	2.6	5.6%
Using the screening tool (AUDIT C)	2.0	0.0%	2.5	25.0%	2.1	5.6%
Explaining the assessment of alcohol use	2.5	7.7%	2.6	20.0%	2.5	11.1%
Giving patients feedback on their alcohol use	2.1	0.0%	2.6	20.0%	2.3	5.3%
Explaining what is meant by low risk drinking	2.1	0.0%	2.2	0.0%	2.1	0.0%
Explaining the consequences and health risks associated with alcohol use	1.9	0.0%	2.2	0.0%	2.0	0.0%
Referring patients in case of dependence or severe alcohol problems	2.8	14.3%	2.4	0.0%	2.7	10.5%
Total mean	2.3	0.0%	2.5	0.0%	2.3	0.0%
N	14		5		19	

^a^ 1 = Very easy, 2 = quite easy, 3 = neither difficult nor easy, 4 = quite difficult, 5 = very difficult.

This is also true for delivering ASBI in general. One noteworthy difficulty or barrier for performing ASBI was the lack of compensation for additional work and the rejection of some patients (Supplementary Table S1). However, too little time for delivering ASBI was viewed as the most significant problem by the physicians. On the other hand, the overall relevance of ASBI was not questioned.

Besides potential barriers, the physicians were also asked about facilitators for conducting alcohol screening and brief intervention/simple feedback (Supplementary Table S2). Here the instruction material provided to the physicians was rated as the most useful facilitator, closely followed by the patient information leaflet which also proved to be helpful in the consultation process.

When asked about the time spent on the screening procedure, the majority of physicians (61.1%) reported an average span of three to 5 minutes per patient (IG: 71.4%, N = 14; CG: 25.0%, N = 4), while one third stated that it took them five to 10 minutes (IG: 21.4%, N = 14; CG: 75.0%, N = 4).

From the patient perspective, nearly all persons who were reached for follow-up confirmed that they had received a personal feedback by the physician (IG: 100.0%; CG: 97.9%) and none of the patients disagreed that the information provided by the physician was consistent. Furthermore, 85.5% of the patients agreed that information about alcohol-related consequence is important for them (IG: 92.9%; CG: 83.3%; χ^2^ = 0.88, *p* = 0.928) and 75.8% agreed that their physician should ask them about their alcohol consumption on a yearly basis (IG: 85.7%; CG: 72.9%; χ^2^ = 2.60, *p* = 0.628) which indicates a high level of acceptance of the measure among the patients.

### Maintenance

The majority of physicians stated that they would integrate ASBI/simple feedback into their future practice (82.4%) (IG: 85.7%; CG: 66.7%). When asked about possible measures to facilitate the future implementation of ASBI/simple feedback, the recommendation to focus the screening on vulnerable and risk groups only, received the highest rate of agreement (Supplementary Table S3), followed by the proposal to let trained nurses conduct the screenings and remunerating physicians for conducting screenings and brief intervention/simple feedback.

## Discussion

This is the first feasibilty trial on ASBI implementation in PHC in Kazakhstan to identify and explore key ASBI implementation determinants and outcomes based on the RE-AIM framework [33]. The study outcomes indicate that screening for alcohol followed by a standardised brief intervention is feasible and can be implemented in PHC settings in Kasakhstan. Part of the feasibility trial was a follow-up evaluation of changes in drinking behaviour after 3 months which represented the effectiveness dimension of the RE-AIM framework. It was the expressed interest of the participating physicians and facilities to evaluate the effect of the screening and brief intervention within the pilot trial. Both approaches, ASBI and the provision of a standard alcohol leaflet can have the potential to achieve a substantial, positive public health impact. By using the RE-AIM framework, the study provides valuable insights into the key determinants of ASBI delivery, which can be used to tailor ASBI implementation strategies and which support the design and implementation of future ASBI trials in Kazakhstan.

One key domain of the RE-AIM framework is the Reach component, the measure to what extent the target population has been reached. Less than half of the invited physicians took part in the training, and only 28% performed the screening. However, among the participating physicians in this trial we found an overall screening rate of 15.7% among eligible PHC patients, which is higher than those found in comparable studies in the field [21, 35]. Interestingly, the screening rate was more than two times higher in the CG (simple feedback plus information leaflet) compared to the IG (formal ASBI plus information leaflet). Considering that the overall number of patients eligible for screening was less than the half in the control arm, one explanation could be, that the PHCU in the control arm had more time to screen their patients. The differences between the groups in experience with alcohol related questions and advice at baseline examination may be due to a misunderstanding among the patients of the two clinics who belonged to the intervention arm. The given study information (and patient consent) may have influenced the answer to this question. However, given the small number of PHCUs in the study and the differences between the PHCUs with regard to numbers of patients per unit, we are cautious to draw respective conclusions. Furthermore, the patients of the CG were younger at baseline and to a lesser extent had an academic degree. The AUDIT-C score among the patients from the IG is significantly lower. (Interestingly, this does not apply to the full AUDIT score.) This can be an important confounder which may limit the comparability of IG and CG. A patient-wise randomisation was not possible in this trial. A future randomised controlled trial with more PHCUs and blockwise randomization instead of clustering is needed to provide further relevant data about alcohol screening (and its effects) in PHC settings in Kazakhstan.

In terms of Effectiveness we found positive changes in AUDIT-C scores among persons with a hazardous or risky drinking pattern at baseline, which corresponds to a moderate effect size of d = 0.72 for the total sample. However, no significant differences in the reduction of hazardous drinking could be found between the groups. Both kinds of intervention—brief face-to-face alcohol intervention plus information leaflet vs. simple feedback plus information leaflet—seem to be effective to reduce the alcohol consumption among PHCUs patients with hazardous drinking in Kazakhstan. Given this, our study outcomes support previous findings that alcohol interventions followed by standardised screening procedures can be very brief [8, 10, 36].

Out of more than one hundred PHC physicians invited to the training sessions, less than one third eventually took part in the study (Adoption). As there were no statistical significant differences between participants and non-participants in terms of gender, work experience, overall job statisfaction, and attitudes towards patients with alcohol use disorders (SAAPPQ), our study outcomes indicate that a respresentative sample of PHC physicians took part. However, not all of the 31 physicians participated in the survey on the implementation of ASBI and the study conditions. This applies to a greater extent to physicians of the control arm which could be a sign of limited study compliance in the CG. Previous studies have shown that training is a key component to increase the ASBI uptake and coverage in PHC settings [37], also in low-middle income countries [21].

With respect of the Implementation dimension of the RE-AIM approach, no major implementation problems in both groups of the participating physicians were observed, with the exception of insufficient time during the patient visit. This particular challenge was also stressed in the focus group discussions, by participating and non-participating physicians alike. Possibly, work overload has also kept individual physicians from attending the training. It may further have been a reason for one PHCU’s decision not to take part in the trial. With regard to the entire project, one could argue that the moderate rate of participating physicians indicates implementation difficulties. However, both interventions were carried out in accordance with the study protocol and the patients’ ratings showed a high acceptance and perceived relevance of both interventions.

Maintenance is the final dimension of the RE-AIM framework which is normally not easy to study within a time-restricted pilot trial. The physicians were asked if they would integrate the procedures of ASBI or simple feedback in their future practice, and the majority agreed to do so. This was even more the case for physicians in the ASBI arm, suggesting that these physicians considered the intervention package as helpful and effective. However, to what extent (or if at all) ASBI will be part of the routine diagnostic procedure in PHC practices cannot be answered by this pilot study.

This study has some limitations. Planned as a pilot cluster-randomised trial the main outcome measure was feasibilty and (to a certain extent) efficacy of ASBI in PHC settings in Kazakhstan. The non-participation of one PHCU in the ASBI arm after randomisation required a higher engagement of the other two units which might have influenced the study outcomes. Within the framework of the present study it was not possible to substitute the non-participating facility, as the total number of public PHCUs in the closer Pavlodar area had already been included in the trial. Furthermore, it has to be considered, that the number of physicians in the control arm was low and the respective outcomes need to be handled with caution. In the study protocol we assumed that 4,000 patients needed to be screened to result in a sample size of 400 patients with a positive AUDIT-C score [29]. Although we reached the expected number of patients eligible for alcohol screening according to the inclusion criteria, only 15.7% of them were actually screened. As a result, the number of patients with positive screening in both groups was low (12.3%), which means that the anticipated number of patients could not be achieved. Furthermore, the follow-up rate among screen positive patients at baseline in the ASBI arm was low. Against the background of the (by definition) short intervention, a follow-up period of 3 months seems comparatively long and is likely to have a negative impact on the participation rate. However, since ASBI is intended to achieve a sustainable change in behavior, the observation period should cover at least a few weeks to reflect such a change [38]. Thus, the effect analysis was based on a smaller number of patients than expected which led to a low statistical power, and it was carried out as a per protocol analysis only [29]. Finally, the results on adoption were based on the responses of fewer than the 112 invited physicians, and data on implementation were only available from 19 out of 31 physicians trained within the study. A general limitation of proving the effectiveness of ASBI (even in a patient-wise randomised study) is the fact that screening itself may have an impact on drinking behaviour and the additional effect of BI may be small and therefore not easily demonstrated statistically in a limited clinical trial.

In this two-arm cluster randomised pilot trial we found that ASBI is feasible, that it can be implemented into PHC settings in Kazakhstan, and that it has positive effects on drinking behaviour if the intervention is accompanied by training and respective supporting material. However, the implementation depends on the willingness and interest of the PHCU and the physicians. Furthermore, the drop-out rate among physicians as well as the non-participation of an entire PHCU indicate that introduction of ASBI requires extensive planning and preparation with stakeholder involvement as well as an adequately financed implementation phase that takes into account the (not only study-related) additional effort. The identified barrier that physicians worked under strong time restraints could be encountered in a future trial by having specifically trained PHC nurses conduct the screening instead of the physicians. Also, a future trial should allow for substituting potential drop outs on the PHCU level with similar facilities. The implementation outcomes measured within the RE-AIM framework form a solid base for a full RCT needed to provide further data on the effectiveness of ASBI compared to brief simple feedback.
