# Supplementary material for: Alcohol Screening and Brief Intervention in Primary Health Care in Kazakhstan—Results of a Cluster Randomised Pilot Study
Source: Int J Public Health. 2022 Oct 10;67:1604803. doi: 10.3389/ijph.2022.1604803 (PMC9588940; doi:10.3389/ijph.2022.1604803)
Supplement: Supplementary file 1 [file DataSheet1.docx]

**Supplementary material, additional tables**

Supplement table 1:

Conducting of alcohol screening and brief intervention/simple feedback during the study. Comparison of intervention and control group (multiple responses, physicians’ perspective) (Germany/Kazakhstan, 2022)

|  | **Intervention** | | **Control** | | **Total** | |
| --- | --- | --- | --- | --- | --- | --- |
| **I found it difficult to deliver ASBI because …** | Mean^a)^ | disagree (4/5) | Mean | disagree (4/5) | Mean | disagree (4/5) |
| There was too little time during the patient visit | 1.8 | 7.7% | 1.2 | 0.0% | 1.6 | 5.6% |
| There was no compensation for this additional work | 2.8 | 23.1% | 3.0 | 0.0% | 2.8 | 16.7% |
| The patients were reluctant to undergo screening | 2.9 | 21.4% | 2.6 | 20.0% | 2.8 | 21.1% |
| There was no obligation made by the administration to carry out this work | 4.6 | 84.6% | 3.3 | 50.0% | 4.3 | 76.5% |
| The entire effort was too high | 3.0 | 15.4% | 3.4 | 40.0% | 3.1 | 22.2% |
| I am not convinced that alcohol screening and brief interventions/ simple feedback have any effect at all | 3.4 | 46.2% | 3.2 | 40.0% | 3.3 | 44.4% |
| I do not want to endanger a good physician-patient relationship by taking about alcohol usage without any good reason | 3.8 | 50.0% | 4.0 | 80.0% | 3.9 | 58.8% |
| I believe that psychiatrists or narcologists shall take care of this | 3.1 | 38.5% | 3.8 | 80.0% | 3.3 | 50.0% |
| Total mean | 3.1 | 14.3% | 3.0 | 40.0% | 3.1 | 21.1% |
| **N** | **14** | | **5** | | **19** | |

^a)^ 1 = Completely agree, 2 = partly agree, 3 = neither agree nor disagree, 4 = partly disagree, 5 = completely disagree.

Supplement table 2:

Facilitators of conducting ASBI during the study. Comparison of intervention and control group (multiple responses, physicians’ perspective) (Germany/Kazakhstan, 2022)

|  | **Intervention** | | **Control** | | **Total** | |
| --- | --- | --- | --- | --- | --- | --- |
| **Facilitators** | Mean^a)^ | disagree (4/5) | Mean | disagree (4/5) | Mean | disagree (4/5) |
| The instruction material was useful | 1.1 | 0.0% | 1.6 | 0.0% | 1.2 | 0.0% |
| It was helpful for the consultation process to be able to give the leaflets to the patients | 1.2 | 0.0% | 2.0 | 20.0% | 1.4 | 5.6% |
| I had sufficient training on how to deliver screening and BI / simple feedback | 1.9 | 0.0% | 2.0 | 25.0% | 1.9 | 5.9% |
| I had enough support and control from my administration | 1.8 | 0.0% | 1.8 | 0.0% | 1.8 | 0.0% |
| I had enough support from the experts during the implementation | 2.1 | 0.0% | 2.2 | 0.0% | 2.1 | 0.0% |
| With increasing duration of the study I found it easier to deliver screening and BI / simple feedback | 1.7 | 0.0% | 2.6 | 40.0% | 1.9 | 11.1% |
| Total mean | 1.6 | 0.0% | 2.1 | 0.0% | 1.7 | 0.0% |
| **N** | **14** | | **5** | | **19** | |

^a)^ 1 = Completely agree, 2 = partly agree, 3 = neither agree nor disagree, 4 = partly disagree, 5 = completely disagree.

Supplement table 3:

Facilitators for future implementation of alcohol screening and brief intervention in primary health care. Comparison of intervention and control group (multiple responses, physicians’ perspective) (Germany/Kazakhstan, 2022)

|  | **Intervention** | | **Control** | | **Total** | |
| --- | --- | --- | --- | --- | --- | --- |
| **Future implementation can be facilitated by …** | Mean^a)^ | disagree (4/5) | Mean | disagree (4/5) | Mean | disagree (4/5) |
| Integrating the screening documents into the standard electronic documentation software for PHC physicians | 1.7 | 8.3% | 2.4 | 20.0% | 1.9 | 11.8% |
| Remunerating physicians for time spent on screening and brief intervention | 1.5 | 0.0% | 2.4 | 0.0% | 1.7 | 0.0% |
| Offering regular trainings on alcohol screening and brief interventions to physicians | 1.8 | 8.3% | 2.0 | 20.0% | 1.8 | 11.8% |
| Integrating the screening tool on alcohol into the national screening program | 1.8 | 7.7% | 2.4 | 20.0% | 1.9 | 11.1% |
| Focusing the screening on vulnerable or risk groups | 1.3 | 0.0% | 2.0 | 0.0% | 1.5 | 0.0% |
| Letting trained nurses conduct the screenings, instead of physicians | 1.4 | 0.0% | 2.5 | 25.0% | 1.6 | 44.4% |
| Legal support of alcohol screening and brief intervention by the Ministry of Health | 1.6 | 0.0% | 2.6 | 40.0% | 1.9 | 58.8% |
| Total mean | 1.5 | 0.0% | 2.4 | 20.0% | 1.7 | 5.3% |
| **N** | **13** | | **5** | | **19** | |

^a)^ 1 = Completely agree, 2 = partly agree, 3 = neither agree nor disagree, 4 = partly disagree, 5 = completely disagree.
